# Research on the resilience of ecological networks from the perspective of ecological security pattern: a case study of Wuhan metropolitan area

**DOI:** 10.1038/s41598-025-29955-7

**Published:** 2025-12-04

**Authors:** Yingchao Zhao, Yucheng Fang, You Zou, Guiyuan Li, Bowen Li

**Affiliations:** 1 School of Civil Engineering, Architecture and Environment, Hubei University of Technology, Wuhan, 430068 China; 2 Key Laboratory of Intelligent Health Perception and Ecological Restoration of Rivers and Lakes, Ministry of Education, Hubei University of Technology, Wuhan, 430068 China; 3 Hubei United Investment Digital Industry Group, Wuhan, 430071 China

**Keywords:** Ecological network resilience, MCR model, Gravity model, Robustness model, Wuhan metropolitan area, Ecology, Ecology, Environmental sciences, Environmental social sciences

## Abstract

Ecological network resilience—the fundamental capacity of ecosystems to maintain functional stability under external disturbances—has emerged as a focal topic in regional ecological security research. This study focuses on the Wuhan Metropolitan Area, employing multi-temporal datasets spanning 2000–2020. First, we developed an ecological security pattern evaluation framework integrating water resources, soil and water conservation, and ecosystem quality to identify ecological source areas. Next, we established a three-dimensional evaluation index system encompassing natural environment, human activities, and physical barriers to generate resistance surfaces. The MCR model was applied to extract ecological corridors and construct the source–corridor pattern. The gravity model was employed to construct ecological networks and analyze their topological structures. Finally, ecological network resilience was assessed through simulated disturbance scenarios. The results indicate that: (1) From 2000 to 2020, the number of ecological source areas was 55, 65, and 54, respectively, exhibiting a “rise–then–decline” trend. Spatially, these areas shifted from scattered to concentrated and contiguous. The network’s core nodes evolved from a decentralized to a highly centralized control structure, increasingly influencing overall network resilience. (2) Over the same period, the number of ecological corridors was 1,485, 2,580, and 1,431, respectively, with primary corridors numbering 41, 89, and 139. These corridors exhibited a spatial pattern of “dense in the south and sparse in the north, dense in the periphery and sparse in the center.” Despite the overall decrease, interaction strength increased, species circulation efficiency improved, and a stable ecological corridor ring gradually formed. (3) The ecological network evolved from incremental expansion to quality- and efficiency-oriented enhancement, ultimately forming an efficient and stable structure. Based on these findings, we identified 8 core nodes, 36 secondary nodes, 29 general nodes, and 86 key ecological corridors in the Wuhan Metropolitan Area, leading to the construction of a composite ecological security pattern characterized as “one screen, three cores, three axes, and multiple networks.” Targeted optimization strategies were proposed to inform the sustainable development of composite ecosystem regions and guide the construction of ecological security patterns.

## Introduction

Under the dual pressures of global climate change and rapid urbanization, the construction of ecological security patterns has become a global priority for sustaining regional development^1^. The “Global Biodiversity Framework beyond 2020”, proposed under the United Nations Convention on Biological Diversity, emphasizes that developing ecological networks is a critical pathway to enhancing ecosystem resilience^2^. Meanwhile, China’s 14th Five-Year Plan incorporates the Yangtze River Economic Belt into the national territorial spatial plan, prioritizing integrated river basin management and systematic ecological restoration to promote coordinated ecological protection and regional development^3^. The Wuhan Metropolitan Area forms a strategic component of the territorial spatial framework in the middle reaches of the Yangtze River. Encompassing the Jianghan Plain, the western Hubei mountains, and the Jianghan water system, it provides diverse natural support for basin-scale ecosystems^4^. Investigating its ecological security pattern and network resilience provides a scientific basis for restoring the “water–land–city” composite ecosystem and enhancing cross-regional ecological connectivity and coordinated restoration.

Ecological security patterns are multidimensional spatial systems integrating ecological sources and corridors to maintain ecosystem stability and the continuity of ecological flows^5,6^. Their theoretical foundation traces back to Manning, who advocated land-use planning aligned with natural systems. In the mid-20th century, concepts such as green corridors and buffer zones emerged in Europe and North America, forming the core spatial elements of ecological planning^7^. More recently, the focus has shifted toward human–environment interactions in the context of urbanization. For example, Esbah applied landscape matrix analysis to assess edge effects on connectivity^8^; Karen employed spatially explicit models to predict biodiversity loss driven by urban expansion^9^; and Hayward used least-cost path analysis to simulate species migration barriers^10^. These diverse perspectives have collectively enriched both the theoretical framework and the practical implementation of ecological security patterns.

Ecological networks are crucial for preserving the integrity and functionality of ecological security patterns^11–14^. Since Tansley introduced the concept of ecosystems, ecological networks have progressively evolved into a central interdisciplinary topic^15^. From Europe’s landscape corridor systems and North America’s green infrastructure concepts^16^ to contemporary construction models centered on “source–corridor”^17,18^, methods for identifying ecological networks have become increasingly refined. For source identification, two main approaches are commonly adopted: direct selection and multi-index comprehensive evaluation^19–21^. The latter emphasizes ecological functions and dynamic changes to a greater extent. For corridor identification, the Minimum Cumulative Resistance (MCR) model is the dominant approach due to its efficiency and visualization advantages; however, it is constrained by the assumption of uniform weighting^22,23^. To improve identification accuracy, recent studies have incorporated impervious surface area (ISA) and nighttime light (NTL) data into resistance surface modification, enabling a more precise representation of human disturbance intensity^24–26^.

Resilience theory originated in engineering^27^ and was introduced into ecology by Holling^28^, who highlighted ecosystems’ capacity to maintain functional stability under disturbances^29–31^. With the advancement of social–ecological system theory, resilience has been redefined as the dynamic adaptability of complex systems, encompassing structural stability, functional continuity, and self-organization^32–34^. In ecological network research, resilience assessment methods have evolved in parallel with theoretical advancements. Early studies relied on single indicators such as connectivity^35,36^. Later, complex network theory supported the development of three frameworks^37^—“scale-density-morphology”, “resistance-adaptability-recovery"^38,39^, and topological characteristics—shifting assessment from a structural focus to integrated structure–function analysis^40,41^. Given ecological networks’ vulnerability to disturbances, research has progressed from static frameworks to dynamic measurements, revealing resilience evolution through disturbance simulations^39,42^. Overall, research on ecological network resilience underpins urban ecological security and fosters interdisciplinary integration^43,44^.

A review of previous studies shows that most research focuses on provinces, cities, and counties, with study areas generally confined to specific administrative boundaries, and little attention given to cross-boundary regions such as urban agglomerations^45,46^. In existing ecological security pattern studies, indicator selection is often narrow, focusing primarily on the intrinsic characteristics of evaluation objects while neglecting diverse measures such as socio-economic factors and ecological space quality^47,48^. Moreover, most studies examine network structures at a single time point, lacking longitudinal analyses across time series and offering limited insights into the dynamic evolution of ecological security patterns and network resilience^47,49^.

Against this backdrop, this study develops a multi-dimensional ecological assessment system using multi-source data from 2000, 2010, and 2020 to systematically identify ecological source areas. It generates ecological corridors using the MCR model to establish a robust source-corridor spatial pattern. Furthermore, it analyzes the topological structure of ecological networks using complex network theory to clarify structural characteristics. Finally, it evaluates ecological network resilience through disturbance scenario simulations to quantify the system’s adaptive capacity under disturbances.

Compared with prior studies, this research offers three main innovations. First, by taking the Wuhan Metropolitan Area as a cross-administrative regional unit, this study transcends the traditional administrative boundary framework and proposes an approach for cross-regional composite ecological research. Second, it integrates diverse datasets from natural, social, and economic domains, introducing innovative indicators such as nighttime light index and PM2.5 to capture interactions between human and natural ecosystems. Finally, using multi-temporal data from 2000, 2010, and 2020, the study examines the spatiotemporal evolution of ecological security patterns and ecological network resilience in the Wuhan Metropolitan Area, thereby elucidating the impacts of urbanization and climate change on ecological networks.

## Materials and methods

### Study area

**Fig. 1:**
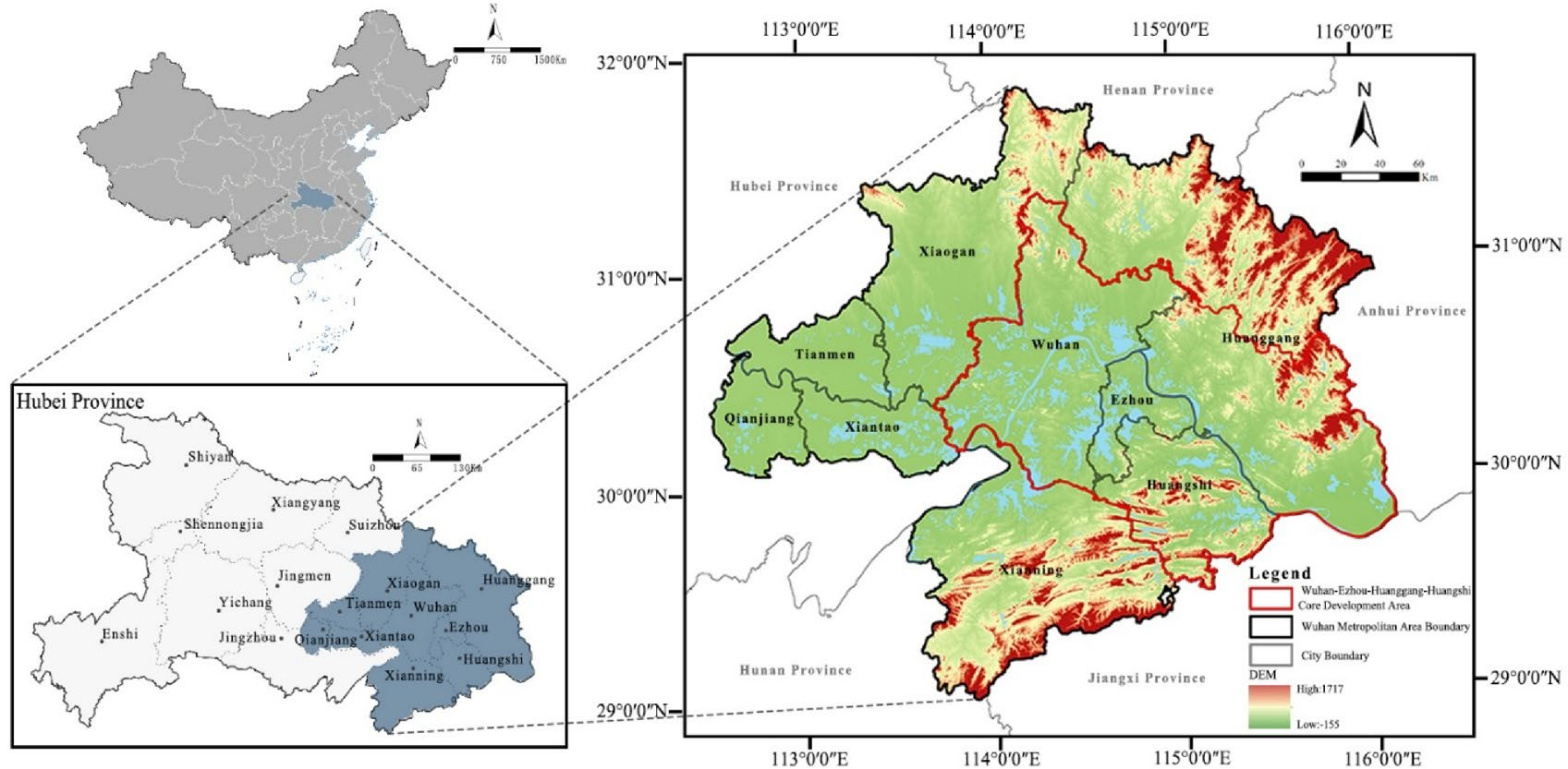
The location of the study area. Maps in this figure were created by the authors using ArcGIS 10.8.1 (Esri, Redlands, CA, USA; https://www.esri.com) and further refined in Adobe Photoshop 2024 (Adobe Inc., San Jose, CA, USA; https://www.adobe.com).

The Wuhan Metropolitan Area lies in the middle reaches of the Yangtze River and the eastern part of Hubei Province (29°30′N–31°36′N, 113°41′E–116°07′E), as illustrated in Fig. 1. It is a core region of the Yangtze River Economic Belt, serving as both an “economic engine” and an “ecological barrier.” Covering one-third of Hubei’s land area and hosting half of its population, the Wuhan Metropolitan Area contributes two-thirds of the province’s GDP, acting as the economic growth engine of the urban agglomeration in the middle Yangtze River Basin. Its ecological foundation comprises an extensive water network, including the Yangtze and Han Rivers, and diverse landforms such as plains, mountains, and hills. Together, these form a “water–land–city” composite ecosystem that serves as a vital corridor for species migration and the flow of matter and energy^50^.

However, rapid urbanization has heightened the region’s ecological sensitivity and vulnerability. Natural barriers such as the Dabie Mountains in the northeast and the Mufu Mountains in the south provide essential regulatory and supporting ecosystem services. The Jianghan Plain in the central-west, with its favorable living conditions, has become a high-demand area for ecosystem services. The spatial and temporal mismatch between ecosystem service supply and demand has further intensified ecological risks in the Wuhan Metropolitan Area. Therefore, the Wuhan Metropolitan Area provides an ideal case for examining ecological security patterns and network dynamics across ecological transition zones, vulnerable areas, and rapidly urbanizing regions. This study addresses the scientific need to reconcile the region’s “ecology–economy” contradiction and represents a practical step toward implementing the ecological civilization strategy of the Yangtze River Economic Belt, offering insights for ecological governance in similar regions worldwide.

### Data source

In this study, we collected multi-source data for the Wuhan Metropolitan Area for the years 2000, 2010, and 2020 to assess its ecological conditions. The dataset comprises natural ecological, economic, and social variables (Table 1). Due to data availability constraints, elevation, slope, soil type, and soil organic matter content were obtained for only one year. However, these variables change minimally over long periods or represent a relatively small proportion of the dataset, exerting little influence on the time-series analysis. The earliest available NPP data were from 2001; therefore, we substituted 2001 data for the year 2000. The most recent GDP data were from 2019, which were used to represent 2020 values. All spatial datasets were resampled to a 1,000 m × 1,000 m resolution.

**Table 1:** Data description.

Category	Data name	Year	Data sources	Resolution
Natural ecological data	DEM	2022	GEBCO(https://www.gebco.net/)	500 m
	Slope	2022	Copernicus Data Space Ecosystem (https://panda.copernicus.eu/panda)	30 m
	River network data	2000,2010,2020	Big Earth Data Science Engineering Project (https://data.casearth.cn/)	
	Land use data	2000,2010,2020	Resources and Environment Science and Data Center (https://www.resdc.cn/)	30 m
	Soil type data	2023	National Cryosphere Desert Data Center (http://www.crensed.ac.cn/portal/)	30 arc-seconds
	Soil organic matter content	1980s	National Tibetan Plateau Data Center (https://data.tpdc.ac.cn/)	1 km
	Soil moisture content	2000,2010,2020	National Tibetan Plateau Data Center (https://data.tpdc.ac.cn/)	1 km
	Annual average precipitation	2000,2010,2020	China Meteorological Data Service Center (http://data.cma.cn/)	1 km
	NDVI	2000,2010,2020	Resources and Environment Science and Data Center (https://www.resdc.cn/)	1 km
	NPP	2001,2010,2020	NASA Earthdata(https://lpdaac.usgs.gov/)	500 m
Economic data	GDP	2000,2010,2019	Figshare(https://info.figshare.com/)	1 km
	NPP/VIIRS, DMSP/OLS	2000,2010,2020	Harvard Dataverse (10.7910/DVN/YGIVCD)	1 km
Social data	Population density	2000,2010,2020	LandScan(https://landscan.ornl.gov/)	1 km
	Road network data	2000,2010,2020	National Geomatics Center of China (https://www.ngcc.cn/)	
	PM2.5	2000,2010,2020	National Tibetan Plateau Data Center (https://data.tpdc.ac.cn/)	1 km

### Methods

**Fig. 2:**
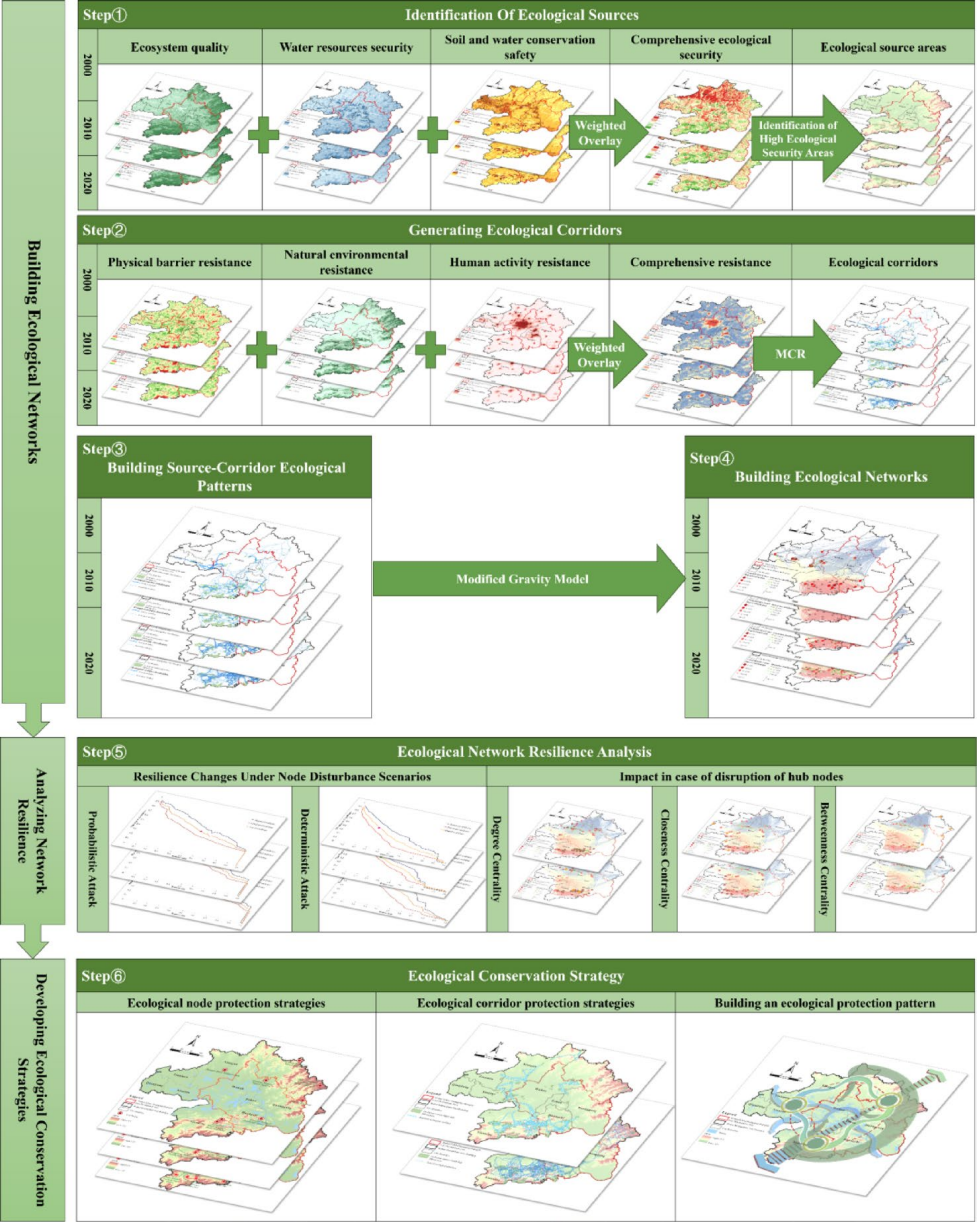
The research framework. Maps in this figure were created by the authors using ArcGIS 10.8.1 (Esri, Redlands, CA, USA; https://www.esri.com) and further refined in Adobe Photoshop 2024 (Adobe Inc., San Jose, CA, USA; https://www.adobe.com).

This study investigates the development and resilience of the ecological network in the Wuhan Metropolitan Area from 2000 to 2020. First, a three-dimensional evaluation index system—comprising ecosystem quality, water resource security, and soil–water conservation—was developed to analyze spatiotemporal variations in ecological security, thereby identifying ecological source areas. A separate three-dimensional index system, incorporating the natural environment, human activities, and physical barriers, was established to analyze the spatiotemporal dynamics of resistance surfaces. Using the identified ecological sources and composite resistance surfaces, the MCR model was applied to extract ecological corridors, thereby constructing a source–corridor spatial framework and assessing its evolutionary characteristics. Subsequently, a gravity model was used to construct the ecological network, followed by an analysis of its topological structure. Furthermore, two disturbance-scenario simulation methods—probabilistic attacks and deterministic attacks—were applied to assess changes in ecological network resilience and the extent of impacts when core nodes were damaged. Finally, optimization strategies were proposed based on the current status of the ecological network in the Wuhan Metropolitan Area (Fig. 2).

#### The identification of ecological sources

Based on previous studies^51–54^ and the ecological characteristics of the Wuhan Metropolitan Area, a three-dimensional indicator system was constructed to assess ecological security, integrating water resource security, soil-water conservation security, and ecosystem quality. Thirteen indicators were selected across these dimensions (Table 2), with weights assigned using the entropy weight method^47,51^. Weighted summation yielded a comprehensive ecological security level. Applying the natural breaks classification, the ecological security pattern was categorized into five tiers: high, relatively high, moderate, relatively low, and low security. Regions classified as high-security zones were identified as ecological source areas for the Wuhan Metropolitan Area.

**Table 2:** Ecological security level evaluation index system.

Target layer	Criterion layer	Index layer	Indicator Effect	Indicator Weight
Ecological security level	Water resource security	River network density	+	0.3251
		Soil moisture content	+	0.0067
		Distance to water sources	+	0.1483
		Annual average precipitation	+	0.0762
	Soil-water conservation security	DEM	-	0.0087
		Slope	-	0.0085
		Soil type	+	0.0996
		Soil organic matter content	+	0.0926
		NPP	+	0.0477
	Ecosystem quality	NDVI	+	0.0086
		Biodiversity index	+	0.0622
		Land use type	+	0.0560
		PM2.5	-	0.0599

1. The biodiversity abundance index is a critical indicator of ecosystem productivity and stability, with higher values reflecting stronger ecological restoration capacity^54^. The formula is defined as^51,55^:
  1 $$\it \:\text{f=(0.5a+0.9f+0.6g+0.8w+0.2c+0.3u}\text{)}\text{/area}$$
  In the equation, the parameters *a*, *f*, *g*, *w*, *c*, and *u* denote the areas of cultivated land, forest land, grassland, water bodies, construction land, and unused land, respectively.
2. Soil type and land use classifications primarily adhere to the Chinese Land Use Classification System and Chinese Soil Classification System published by the Chinese Academy of Sciences, supplemented by related studies^56^. Based on their relative ecological importance, soil and land use types are classified into five distinct security-level land use categories (Table 3).
  **Table 3:** Classification criteria for soil types and land use.

	High security level	Relatively high security level	Moderate security level	Relatively low security level	Low security level
Ecological source resistance value	1	2	3	4	5
Land use types	Water bodies	Forest land	Grassland	Cropland	Built-up and unused land
Soil types	Turfy soil, cryogenic frozen soil, chernozem, phaeozem, kastanozem	Meadow soil, skeletal soil, irrigated desert soil, gray desert soil, gray desert soil	Gray-brown desert soil, newly deposited soil	Saline soil, alluvial soil, peat soil, grey-cinnamon soil	Residual saline soil, lithosol, aeolian sandy soil

#### The construction of the resistance surface

The resistance surface quantifies the spatial impediments encountered by ecological species and biological flows during movement, with influencing factors primarily categorized into human activities and natural environmental conditions^51^. Drawing on prior research^36,57–59^ and data availability, this study constructed a three-dimensional indicator system encompassing natural environment, human activities, and physical barriers. Ten indicators^17,18,20,41,60^—including road network density—were selected to characterize the magnitude of resistance imposed by natural conditions and anthropogenic factors on ecological processes (Table 4).

**Table 4:** Resistance surface evaluation indicator System.

Target layer	Criterion layer	Index layer	Indicator effect	Indicator weight
Resistance surface	Natural environment	DEM	+	0.1175
		Slope	+	0.1174
		NDVI	-	0.0101
	Human activities	Population density	+	0.1405
		GDP	+	0.2748
		Nighttime light intensity	+	0.2176
	Physical barriers	Land use type	+	0.0100
		Road network density	+	0.0498
		Water network density	-	0.0009
		Distance to water bodies	+	0.0614

#### Ecological corridor generation

The MCR model was applied to generate ecological corridors based on the identified ecological sources and composite resistance surfaces^47,61^. The MCR model calculates the least-cost paths between ecological sources by minimizing the cumulative resistance values across the landscape^62^. The formula is defined as:

2 $$\it \:\text{MCR=f}\text{min}{\sum\:}_{\text{j=n}}^{\text{\:i=m}} (\text{Dij} \times \text{Ri})$$

*f* is a monotonically increasing function reflecting the positive correlation between the minimum cumulative resistance at any spatial point, its distance to the source area, and the characteristics of the landscape matrix. *D*_*ij*_ represents the Euclidean spatial distance from ecological source *j* to landscape matrix unit *i*. *Ri*​ denotes the resistance coefficient of landscape matrix unit *i* for species movement.

#### Ecological network construction

The gravity model is employed to quantitatively assess the mutual influence between spatial elements at a given scale, particularly for evaluating the interaction forces between ecological sources^22,47^. Stronger interaction forces between sources indicate higher importance of the corresponding ecological corridor^63,64^. This study refined the gravity model by integrating biological flow resistance and ecological source-corridor theory, constructing an ecological network mapping framework for analyzing topological structure and resilience. The model is defined as:

3 $$\it \:\text{Gij}\text{=}{{\text{L}}^{\text{2}}}_{\text{max}}\text{In}\left({\text{S}}_{\text{i}}\right)\text{In}\left({\text{S}}_{\text{j}}\right)/\left({{\text{L}}^{\text{2}}}_{\text{ij}}{\text{P}}_{\text{i}}{\text{P}}_{\text{j}}\right)$$

*Gij​* represents the interaction force between source *i* and source *j*. *L*_*max*_​ denotes the maximum cumulative resistance value among all regional corridors. *S*_*i*_ and *S*_*j*_ are the areas of source *i* and source *j*, respectively. *Lij*​ is the cumulative resistance value of the corridor between source *i* and *j*. *P*_*i*_​ and *P*_*j*_ are the resistance values of sources *i* and *j*.

#### Ecological network topological structure analysis

Matching, hierarchy, transmission, and agglomeration are critical for evaluating ecological network structural characteristics^47^. Drawing on established methodologies^65^, this study employed degree distribution, degree correlation, average path length, and clustering coefficient to analyze network hierarchy, matching, transmission, and agglomeration (Table 5). Additionally, degree centrality, betweenness centrality, and closeness centrality were applied to dissect the topological structure of the ecological network^66^.

**Table 5:** Topological structure analysis indicators for ecological networks.

Target	Index	Method	Expression
Network topology	Centrality	Degree centrality	$\it \:{\text{C}}_{D}\left(\text{i}\right)\text{}{=}\sum\:_{\text{j=1}}^{\text{n}}\text{Xij}\text{,}{\text{C}}_{\text{D}}^{{\prime\:}}\left(\text{i}\right)={\text{C}}_{\text{n}}\left(\text{i}\right)/(n-1)$ $\it \:{\text{C}}_{D}\left(\text{i}\right)\text{}$is the absolute centrality of node *i*;$\it \:\text{}\text{X}\text{ij}$ is the strength of connection between node *i* and node *j*;$\it \:\:{\text{C}}_{\text{D}}^{{\prime\:}}\left(\text{i}\right)$ is the relative centrality. Centrality represents the quantification of the importance of a node to the ecological network, the greater the centrality, the more functional the point is.
		Betweenness centrality	$\it \:{\text{C}}_{R}\left(\text{i}\right)\text{=}{\sum\:}_{\text{j}}^{n}{\sum\:}_{k}^{\text{n}}\text{gjk}\left(\text{i}\right)/\text{gjk}$, j < k, j ≠ k≠i $\it \:{\text{C}}_{R}\left(\text{i}\right)$denotes the mediator centrality degree;$\it \it \:{\text{g}}_{jk}\text{}$represents the number of shortest paths that exist between node *j* and node *k*. The mediacy degree characterizes the role of the node as a bridge in the ecological network.
		Closeness centrality	$\it \:{\text{C}}_{\text{c}}\left(\text{i}\right) {=(n-1)/}{\sum\:}_{\text{j}-1, \text{j} \ne \text{i}}^{\text{n}}{\text{d}}_{\text{ij}}$ $\it \:{\text{C}}_{\text{c}}\left(\text{i}\right)\:$denotes the proximity centrality of node *i*;$\it \:{\text{d}}_{\text{ij}}\text{}$refers to the shortest path length from node *i* to node *j*; *n* refers to the total number of nodes in the network. Closeness centrality focuses on the global reachability, which measures the closeness of nodes to the whole network.
	Tightness	Network density	$\it {\text{D}} = {\text{m}}/\left[ {{\text{n}} \times ({\text{n}} - 1)} \right]$ *D* denotes network density; *m* denotes the number of actual relationships; *n* denotes the number of nodes. Network density intuitively reflects the closeness of connections between nodes in an ecological network.
	Hierarchy	Degree distribution	$\it \:\text{ln}{\text{C}}_{\text{i}}\text{=}\text{lnK+aln}{\text{C}}_{\text{i}}^{{\prime\:}}\text{,}{\text{}\text{H}}_{\text{N}}\text{=}{\text{C}}_{\text{m}}\text{/}{\text{C}}_{\text{m}}$ $\it \:{\text{C}}_{\text{i}}\text{}$denotes the degree of node *i*; $\it \:{\text{C}}_{\text{i}}^{\prime\:}\text{}$denotes the bit order of the degree of node *i*; *K* is a constant term; *a* is the slope and *a* < 0. A larger slope indicates a more pronounced hierarchy in the network.
	Transitivity	Average path length	$\it \:\text{L=}\frac{\text{1}}{\text{n}\left(\text{n-1}\right)}\sum\:_{\text{j=1}}^{\text{n}}{\text{d}}_{\text{ij}}$ *L* denotes the average path length, which represents the average value of the distance $\it \:{\text{d}}_{\text{ij}}$ between any two nodes in the network, reflecting the overall connectivity and information dissemination rate of the network.

#### Ecological network resilience analysis

To quantitatively evaluate ecological network resilience, it is essential to establish appropriate measurement indicators^32,67^. This study adopts a dual perspective, incorporating functional and structural resilience, and selects four indicators—agglomeration, connectivity, transmissibility, and diversity (Table 6)—to construct the ecological network resilience assessment system^41,68^. Ecological networks are primarily subjected to two types of simulated attacks: probabilistic and deterministic^41,66^. Python software was used to simulate changes in network resilience under these attack scenarios. In deterministic attack simulations, ecological nodes—the basic units of the network derived from simplified ecological source areas using the improved gravity model—are first ranked by importance. The resilience of the network is then evaluated after sequential removal of core nodes, which are defined as nodes with relatively high comprehensive centrality identified through complex network topological analysis and crucial for maintaining network stability. In probabilistic failure simulations, nodes are not ranked. Changes in network resilience are measured by randomly deleting a given number of nodes.

**Table 6:** Topological structure analysis indicators for ecological networks.

Target	Index	Method	Expression
Functional resilience	Agglomeration^57^	The average clustering coefficient	$\it \:{\text{C}}_{\text{i}}\text{=}\frac{\text{2}{\text{ED}}_{\text{i}}}{{\text{k}}_{\text{i}}\text{(}{\text{k}}_{\text{i}}\text{-1)}}\text{,A=}\frac{\sum\:_{\text{i=1}}^{\text{n}}{\text{C}}_{\text{i}}}{\text{n}}$ *A* is the average clustering coefficient, $\:{k}_{i}$ is the degree of node *i*, $\:{C}_{i}$ is the local clustering coefficient of node *i*, $\:{ED}_{i}$ is the actual number of edges between node *i* and its neighboring nodes, and *n* is the total number of nodes in the network.
	Connectivity^35^	The mean connectivity degree	$\:\text{K=}\sum\:_{\text{i}}{\text{k}}_{\text{i}}\text{/n}$ *K* is the mean connectivity degree of the network; $\:{\text{k}}_{\text{i}}$ is the degree of node *i*; and *n* is the total number of nodes in the network.
Structural resilience	Transmissibility^29^	The network efficiency	$\it \:\text{E=}\sum \limits_{{\text{i}} \ne {\text{j}} \in {\text{G}}} \frac{\text{1}}{{\text{d}}_{\text{ij}}}\text{/n(n-1)}$ *E* is the network efficiency, where 0 ≤ E ≤ 1;$\it \:{\text{d}}_{\text{ij}}$is the shortest distance between node *i* and node *j* in the network; *n* is the total number of nodes in the network; and *G* is the set of nodes in the network.
	Diversity^69^	The average number of independent paths	$\it \:\text{V=}\sum \limits _{{\text{i}} \ne {\text{j}} \in {\text{G}}} {\text{n}}_{\text{ij}}\text{/n(n-1)}$ *V* is the average number of independent paths; $\it \:{\text{n}}_{\text{ij}}\:$is the number of independent paths between node *i* and node *j* in the network; *n* is the total number of nodes in the network; and *G* is the set of nodes in the network.

The above indicators describe the resilience of the ecological network from four different perspectives. To obtain an overall resilience score by integrating these diverse values, it is necessary to standardize the indicators and then calculate the structural resilience$\:\:{S}_{i}$, functional resilience $\:{F}_{i}$, and overall resilience $\:{R}_{i}$ of the EN using equal weights^41,68^.

4 $$\it \:{\text{S}}_{\text{i}}\text{=}\left(\text{N}{\text{A}}_{\text{i}}\text{+N}{\text{K}}_{\text{i}}\right)\text{/2}$$

5 $$\it \:{\text{F}}_{\text{i}}\text{=}\left(\text{N}{\text{E}}_{\text{i}}\text{+}\text{N}{\text{V}}_{\text{i}}\right)\text{/2}$$

6 $$\it \:{\text{R}}_{\text{i}}\text{=}\left({\text{S}}_{\text{i}}\text{+}{\text{F}}_{\text{i}}\right)\text{/2}$$

where $\it \:\text{N}{\text{A}}_{\text{i}}$ represents the normalized agglomeration, $\it \:\text{N}{\text{K}}_{\text{i}}$ represents the normalized connectivity, $\it \:\text{N}{\text{E}}_{\text{i}}$ represents the normalized transmissibility, and $\it \:\text{N}{\text{V}}_{\text{i}}$ represents the normalized diversity.

## Results

### Coupling relationship between ecological security level and resistance surface

**Fig. 3:**
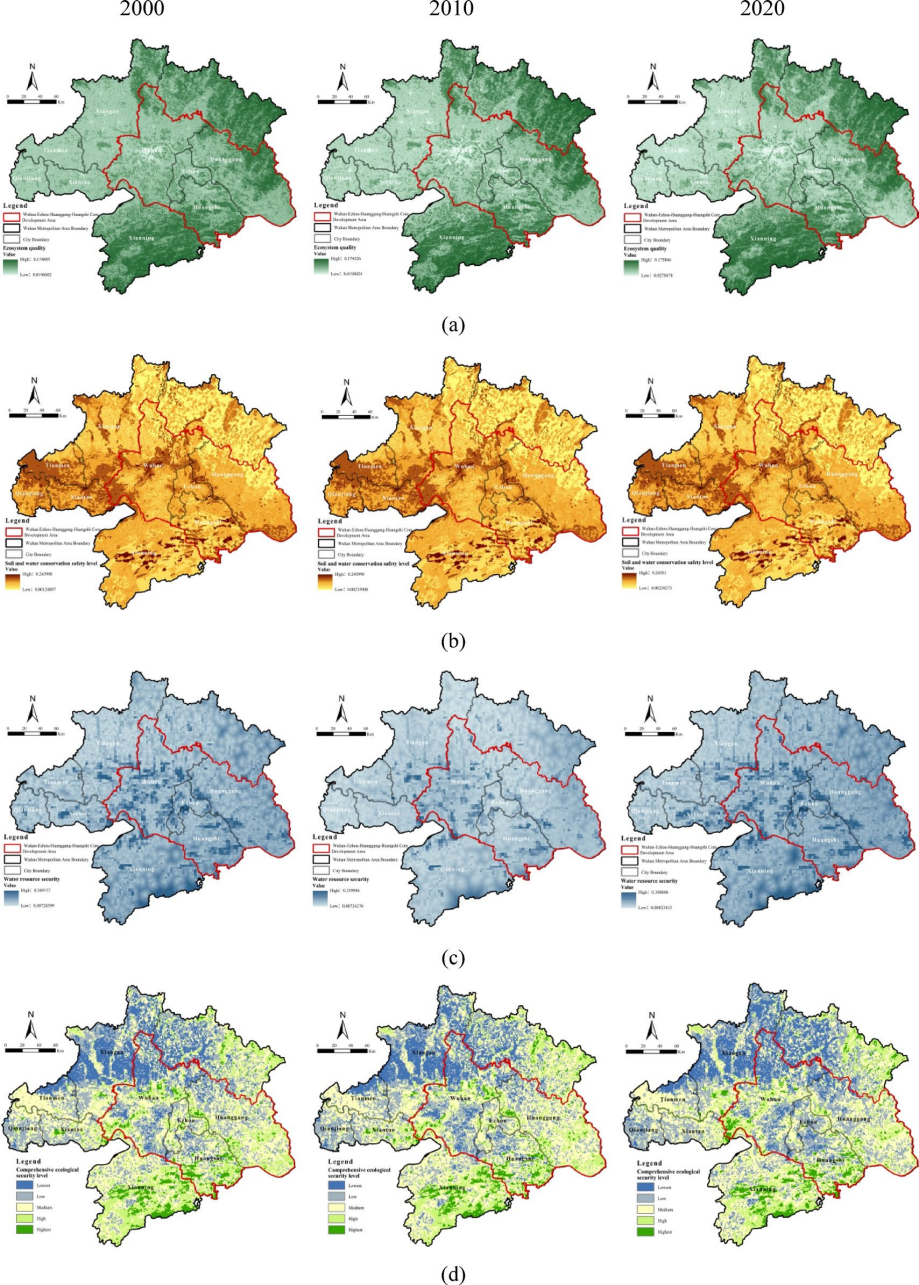
Spatiotemporal evolution map of ecological security levels: (**a**) ecosystem quality; (**b**) soil and water conservation safety; (**c**) water resources security; (**d**) comprehensive ecological security. Maps in this figure were created by the authors using ArcGIS 10.8.1 (Esri, Redlands, CA, USA; https://www.esri.com) and further refined in Adobe Photoshop 2024 (Adobe Inc., San Jose, CA, USA; https://www.adobe.com).

**Fig. 4:**
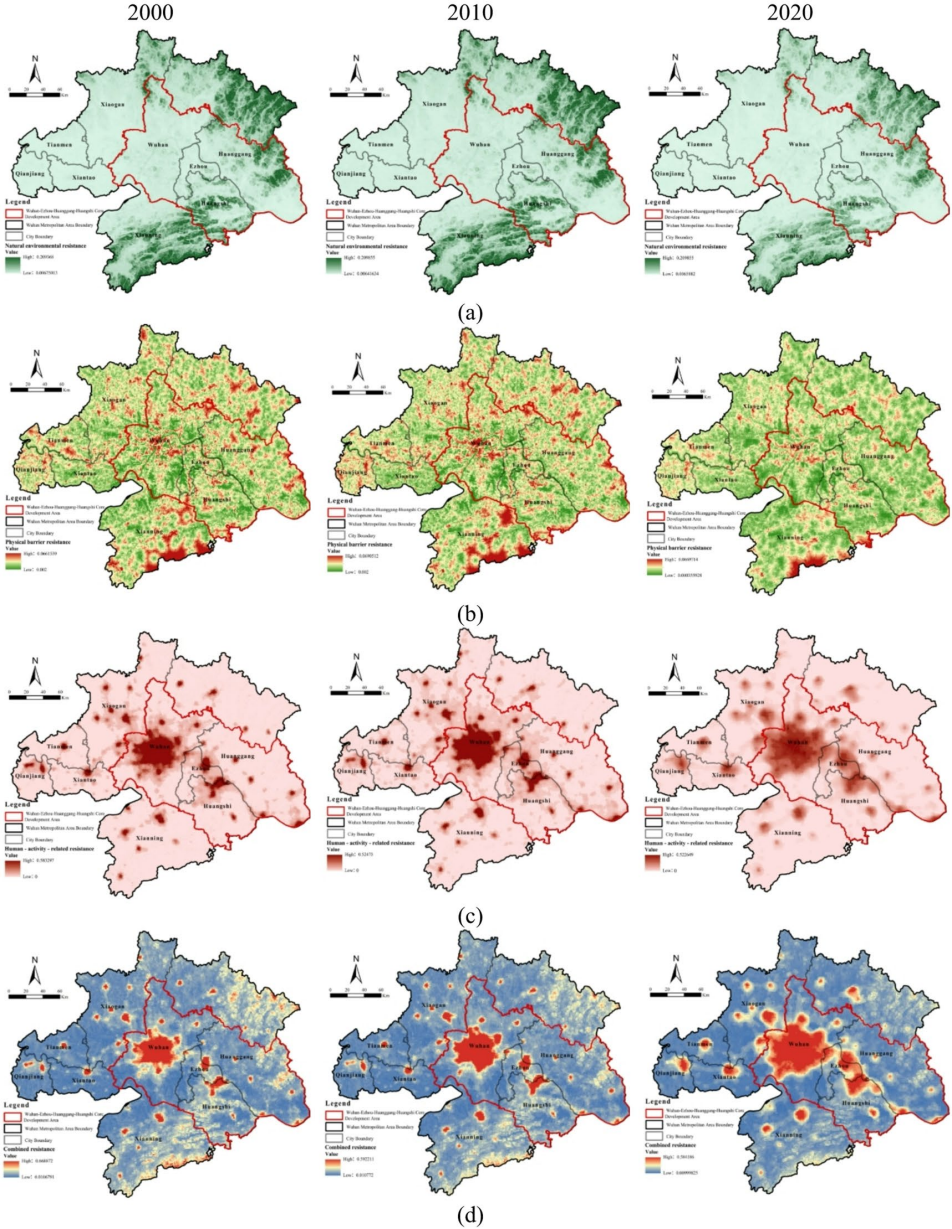
Spatiotemporal evolution map of ecological security levels: (**a**) natural environmental resistance; (**b**) physical barrier resistance; (**c**) human activity resistance; (**d**) comprehensive resistance. Maps in this figure were created by the authors using ArcGIS 10.8.1 (Esri, Redlands, CA, USA; https://www.esri.com) and further refined in Adobe Photoshop 2024 (Adobe Inc., San Jose, CA, USA; https://www.adobe.com).

Using the previously constructed ecological security evaluation index system and resistance surface index system, we quantified and analyzed the ecological security levels and resistance of each dimension in the Wuhan Metropolitan Area. The results are presented in Figs. 3 and 4.

### Single-factor analysis

From 2000 to 2020, the overall ecological security level in the Wuhan Metropolitan Area exhibited an increasing trend, although temporal evolution patterns and spatial distributions varied among ecological dimensions. Ecosystem quality followed a U-shaped trajectory, initially declining and subsequently increasing, whereas soil and water conservation and water resource safety improved steadily, together forming the core support for regional ecological function restoration. Spatially, high-security areas were primarily located in the northeastern Dabie Mountains, southern Mufu Mountains, and lake–wetland zones. The central and western plains exhibited relatively low ecological security, displaying an overall gradient of “high at the edges and lo w in the core.”

1. Ecosystem quality exhibited a U-shaped trend (Fig. 3a), initially declining due to urban expansion, vegetation fragmentation, and pollution, but gradually recovering after 2010 under ecological restoration policies. Spatially, the northeastern Dabie Mountains and southern wetlands maintained relatively high ecological quality, whereas the central and western Jianghan Plain showed lower quality due to intensive urban and agricultural land use. Implementation of the ecological red line policy further intensified regional differentiation.
2. Soil and water conservation capacity steadily improved (Fig. 3b), largely driven by policy interventions such as land consolidation and vegetation restoration. Central and western plains showed notable improvements, whereas northeastern and southern mountainous areas continued to experience soil erosion due to steep slopes and fragile soils. Despite these natural constraints, “sponge city” initiatives mitigated surface runoff and enhanced soil–water stability.
3. Water resource safety continued to strengthen (Fig. 3c), with high-value areas concentrated along the Yangtze River main stem and surrounding lake wetlands. Wetland restoration projects enhanced regional water supply capacity. Nevertheless, inland regions remained under considerable resource pressure due to uneven precipitation and excessive groundwater extraction, highlighting the tension between ecological carrying capacity and human demand.

Regarding the resistance of each dimension from 2000 to 2020, the Wuhan Metropolitan Area exhibited structural diversity and dynamic evolution. The three resistance factors displayed distinct temporal and spatial trends: natural environmental resistance increased steadily, physical barrier resistance intensified, while human activity resistance, although declining numerically, expanded spatially. These factors were spatially intertwined and overlapped, collectively forming an ecological interference pattern characterized by mutual restraint between the “core” and “periphery.”

1. Natural environmental resistance gradually increased, with high-value areas concentrated in the northeastern Dabie Mountains and southwestern Mufu Mountains (Fig. 4a). This region, characterized by steep terrain, high soil moisture, and dense vegetation, was further strengthened by policy-driven vegetation restoration. In contrast, the central and western Jianghan Plain, with flat terrain and intensive human activity, consistently exhibited low resistance, with only local high-value spots in hilly areas.
2. Physical barrier resistance intensified, creating linear high-resistance zones along rivers and scattered high-value points in lake and wetland areas (Fig. 4b). Urban expansion and transportation network development in the Jianghan Plain disrupted ecological connectivity. High resistance was mainly observed in water network–dense regions, whereas urban cores exhibited aggregated moderate resistance due to dense infrastructure.
3. The spatial extent of human activity resistance expanded, although overall intensity declined (Fig. 4c). In the early stage (2000–2010), economic growth and population concentration increased resistance values. Later, resistance declined due to policy interventions, technological advancement, and industrial restructuring. Spatially, Wuhan’s central urban area formed the high-resistance core, whereas central areas of other cities constituted multiple secondary high-value points.

### Coupling relationship analysis

Between 2000 and 2020, the comprehensive ecological security level in the Wuhan Metropolitan Area increased overall, whereas the comprehensive resistance surface declined, indicating continuous improvement in regional ecological quality. However, the spatial distribution of both indicators remained uneven. Generally, areas with high ecological security coincided with regions of low resistance, whereas high-resistance zones corresponded to ecologically fragile areas. This pattern reflects the spatial coupling between ecological protection effectiveness and human disturbance pressure.

The comprehensive ecological security level increased over time, with high-value zones concentrated in the northeastern and southern regions and forming linear distributions along the Yangtze River in the central area (Fig. 3d). National strategies—including Returning Farmland to Forests/Wetlands, High-Standard Farmland Construction, and Yangtze River Protection—synergistically improved soil–water conservation, ecosystem quality, and water security. Spatial heterogeneity reflected interactions between natural and anthropogenic factors. The southern Mufu Mountains and northeastern Dabie Mountains provided key biodiversity and multi-layered ecological security, whereas central Yangtze cities formed linear ecological belts through riparian restoration. Persistent vulnerabilities in the central-western areas, caused by historical pollution and urban encroachment, highlighted ongoing ecological challenges during regional development.

Comprehensive resistance declined over time and was spatially organized into a single-core, multi-scatter pattern centered on Wuhan and eight municipal urban clusters (Fig. 4d). Despite decreasing resistance magnitudes, their spatial influence expanded. Wuhan’s central urban area exhibited peak resistance due to its strategic location at the Yangtze–Han River confluence, economic dominance, and dense lake systems, including East Lake and Tangxun Lake. Moderate-resistance areas were clustered in the northeastern Dabie Mountains and southern Mufu Mountains, where natural environmental resistance remained high.

In summary, a strong spatial coupling exists between comprehensive ecological security and comprehensive resistance in the Wuhan Metropolitan Area. Improvements in ecological security primarily occurred in areas with favorable natural conditions, effective policy interventions, and moderate or declining resistance. In contrast, high-resistance areas remain critical vulnerabilities for ecological governance. Future efforts should focus on enhancing ecological restoration and management in these high-resistance regions to optimize the overall ecological security pattern and promote coordinated regional development.

### Spatiotemporal evolution characteristics of ecological sources areas

From 2000 to 2020, ecological source areas were identified based on high comprehensive ecological security levels, considering natural features—such as dense rivers, lakes, and ports—and anthropogenic pressures, including urbanization. We also referred to established methods to determine the minimum area threshold for ecological source patches^68,70,71^. To maintain ecosystem integrity and key functions, small and scattered patches were excluded, retaining only relatively large contiguous areas. Finally, contiguous patches exceeding 1,000 hectares were designated as ecological sources. Analysis identified 55 core ecological sources in 2000, with an average area of 3,554 ha; 65 sources in 2010 (mean 3,255 ha); and 54 sources in 2020 (mean 3,746 ha) (Table 7). Additionally, in 2020, 73 ecologically significant patches exceeding 500 ha were recorded.

**Table 7:** Interannual variation of ecological source areas.

	2000	2010	2020
Quantity/pc	55	65	54
Average area/ha	3554	3255	3746

**Fig. 5:**
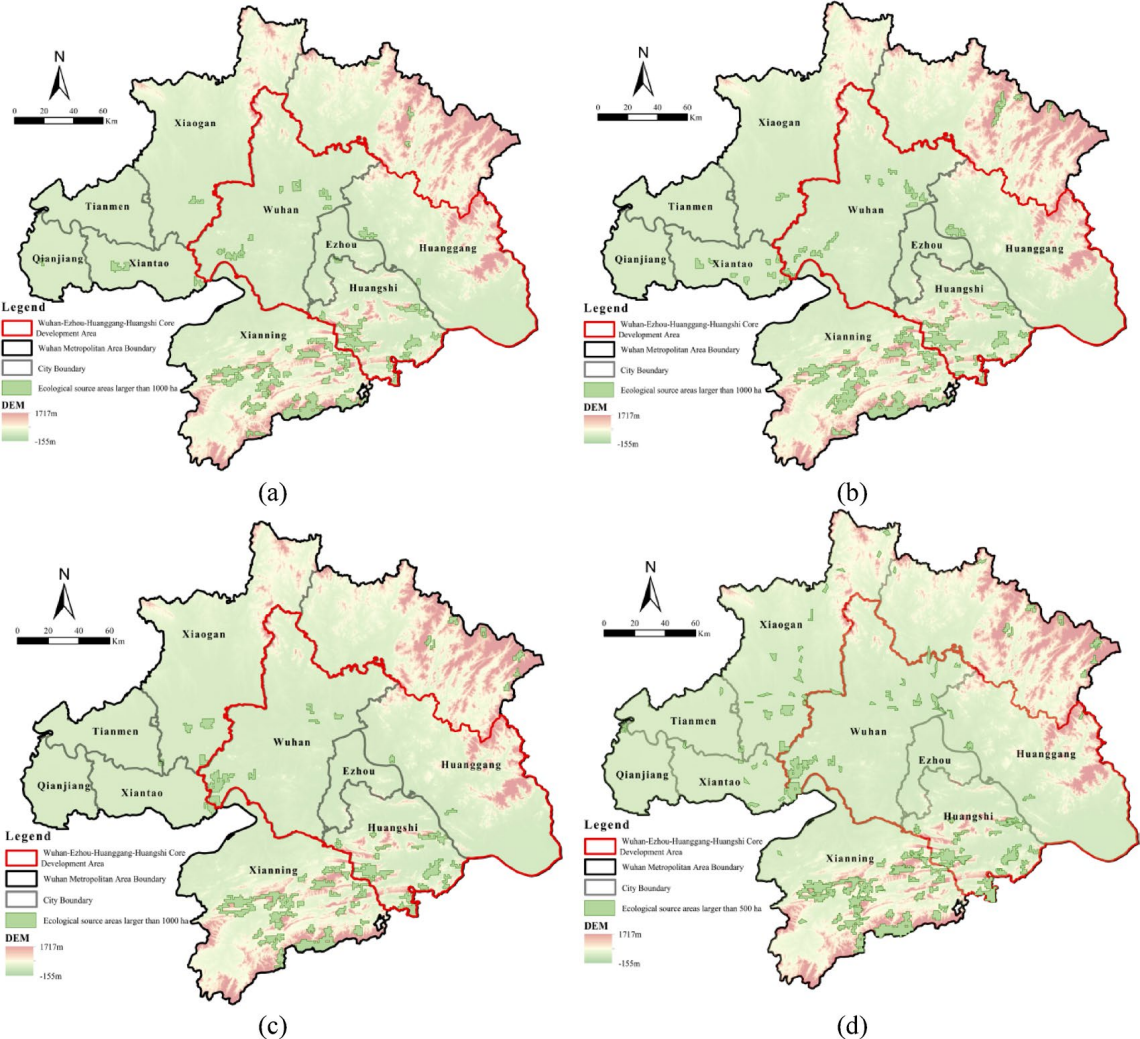
Spatial and temporal evolution of ecological sources: (**a**) ecological source areas (> 1000 ha), 2000; (**b**) ecological source areas (> 1000 ha), 2010; (**c**) ecological source areas (> 1000 ha), 2020; (**d**) ecological source areas (> 500 ha), 2020. Maps in this figure were created by the authors using ArcGIS 10.8.1 (Esri, Redlands, CA, USA; https://www.esri.com) and further refined in Adobe Photoshop 2024 (Adobe Inc., San Jose, CA, USA; https://www.adobe.com).

Between 2000 and 2020, ecological sources larger than 1,000 ha transitioned from fragmented to clustered distributions, with numbers increasing during the early stage (2000–2010) and declining in the later stage (2010–2020) (Fig. 5). The early-stage increase was attributed to forest and wetland restoration in the Dabie Mountains and Liangzi Lake, implemented through the Grain-to-Green Program and Yangtze Shelterbelt Project. During the later stage, ecological redline policies consolidated fragmented patches into two main clusters—the Dabie-Mufu Mountain barrier and Liangzi-Futou Lake wetlands—while urban expansion in the Jianghan Plain eliminated small sources, enhancing peripheral clustering. Spatially, ecological sources were concentrated in southern and western regions. Xianning’s mountainous terrain and supportive policies enabled broad coverage, while Huangshi’s Mufu Hills and western lakes, combined with varied topography, sustained multiple patches. In contrast, northern and central areas suffered fragmentation due to human pressures.

In summary, the Wuhan Metropolitan Area’s ecological sources shifted from fragmented to clustered distributions. Early-stage restoration policies increased the number of large patches, while later-stage ecological redline policies consolidated sources and urban expansion reduced small patches. Sources remain concentrated in southern and western regions, highlighting areas critical for ecological protection.

### Classification and spatiotemporal evolution characteristics of ecological corridors

**Fig. 6:**
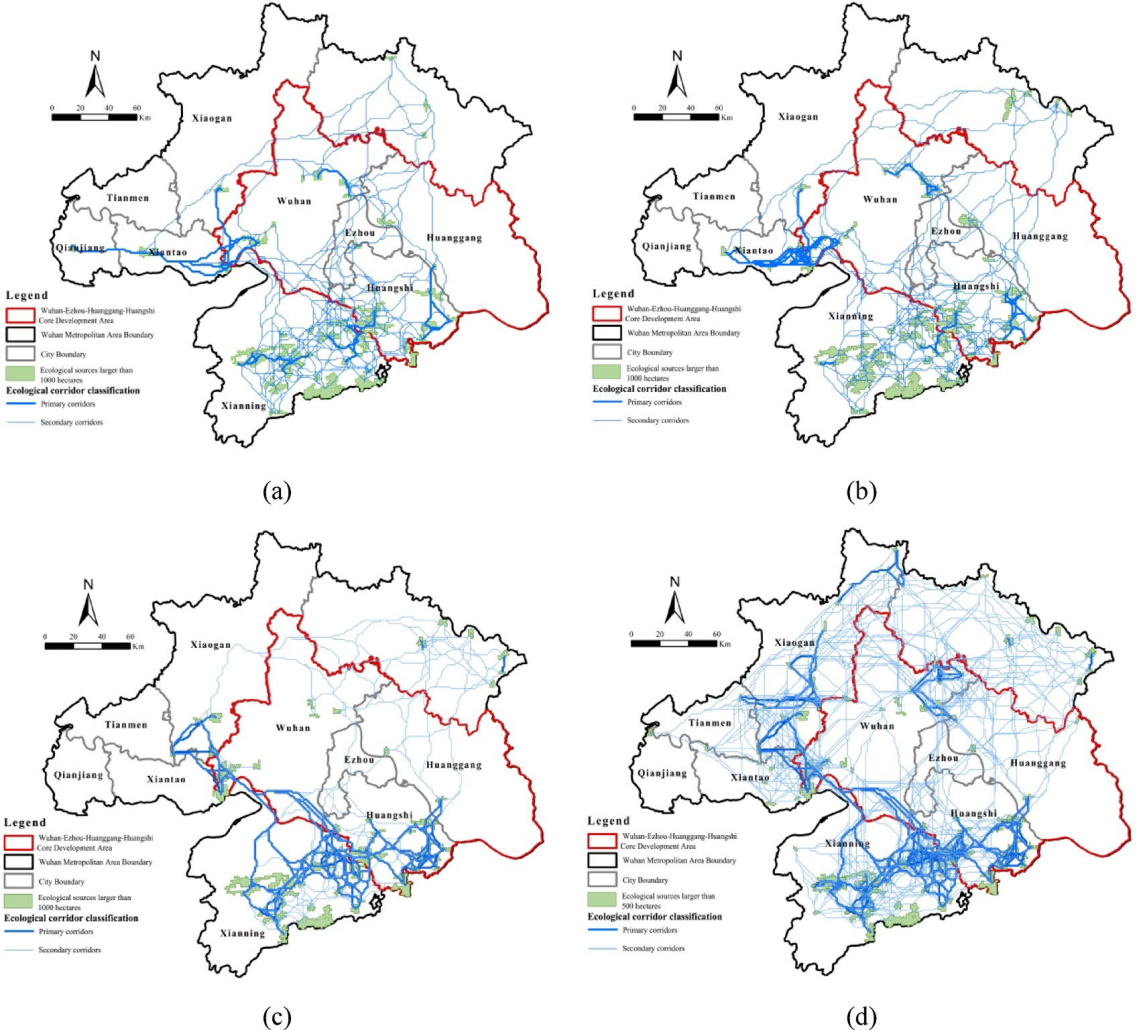
Spatial and temporal evolution of source-corridor patterns: (**a**) source-corridor patterns (> 1000 ha), 2000; (**b**) source-corridor patterns (> 1000 ha), 2010; (**c**) source-corridor patterns (> 1000 ha), 2020; (**d**) source-corridor patterns (> 500 ha), 2020. Maps in this figure were created by the authors using ArcGIS 10.8.1 (Esri, Redlands, CA, USA; https://www.esri.com) and further refined in Adobe Photoshop 2024 (Adobe Inc., San Jose, CA, USA; https://www.adobe.com).

Ecological corridors were generated using the MCR model, connecting the identified ecological sources. Corridors were classified into primary and secondary types according to source interaction strength, with higher interaction indicating greater ecological importance (Fig. 6).

**Table 8:** Interannual variation of ecological corridors.

	2000	2010	2020
Total number of corridors/count	1485	2580	1431
Primary corridors/count	41	89	139

Between 2000 and 2020, ecological corridors in the Wuhan Metropolitan Area evolved from early-stage quantitative expansion to later-stage optimization of key corridors. Spatially, corridors exhibited peripheral density, central sparsity, and relative vulnerability in the northeastern region. The ecological network formed by contiguous patches larger than 1,000 ha displayed dynamic connectivity changes over the study period (Table 8). In 2000 (Fig. 6a), 1,485 corridors, including 41 primary routes, were concentrated in western and southern zones, reflecting robust habitats supporting frequent species migration. By 2010 (Fig. 6b), corridor numbers surged to 2,580 with 89 primary routes, though anthropogenic pressures reduced connectivity in southern Xianning and western Qianjiang. By 2020 (Fig. 6c), corridors declined to 1,431 but prioritized 139 primary routes, concentrated in southern and northeastern sectors through rehabilitation efforts. Improved habitat connectivity facilitated species movement between central-western and southern regions, reflecting policy-driven ecological recovery despite prior fragmentation. In 2020, the ecological network including sources larger than 500 ha consisted of 2,628 corridors, 225 of which were primary. The network formed a consolidated annular configuration, characterized by dense peripheral corridors and sparse central regions (Fig. 6d). Structural vulnerability appeared in the northeastern region, where geomorphic limitations and human activities isolated core nodes and lowered connectivity. Targeted ecological interventions are necessary to bridge connectivity gaps and strengthen overall network cohesion.

In summary, ecological corridors in the Wuhan Metropolitan Area evolved from early-stage quantity expansion to later-stage optimization. Connectivity improved in central-western and southern regions, while northeastern vulnerabilities persisted. Rehabilitation policies increased the number and quality of primary corridors, highlighting priority areas for ecological network management.

### Topological characteristics of the ecological network in the Wuhan metropolitan area

The ecological network was simplified into an undirected, unweighted topological structure using a modified gravity model. Node centrality metrics—degree, closeness, and betweenness—were calculated and weighted using the entropy method to determine overall node importance. Cluster analysis grouped nodes into five hierarchical tiers based on significance and interaction intensity, allowing systematic evaluation of ecological connectivity.

Between 2000 and 2020, the ecological network’s topology became tighter, with stabilized clustering, optimized hierarchy, and efficient transitivity (Table 9). Network density and clustering coefficient increased significantly. Core nodes transitioned from a scattered distribution to concentrated clusters in the southern region. Degree distribution initially increased and later slightly declined. Core node arrangement shifted from expansion to optimization, while network hierarchy underwent a strengthening–balancing adjustment. Average path length decreased initially and then slightly rebounded. Despite minor reductions in transmission efficiency due to network expansion, overall efficiency remained high, and global transmission functionality stabilized.

**Table 9:** Evolution of network topological characteristics.

Index	Method	2000	2010	2020
Tightness	Network density	0.0276	0.0428	0.0971
Agglomeration	Clustering coefficient	0.597	0.690	0.724
Hierarchy	Degree distribution	26.9818	32	27.0189
Transitivity	Average path length	1.5508	1.4910	1.5269

**Fig. 7:**
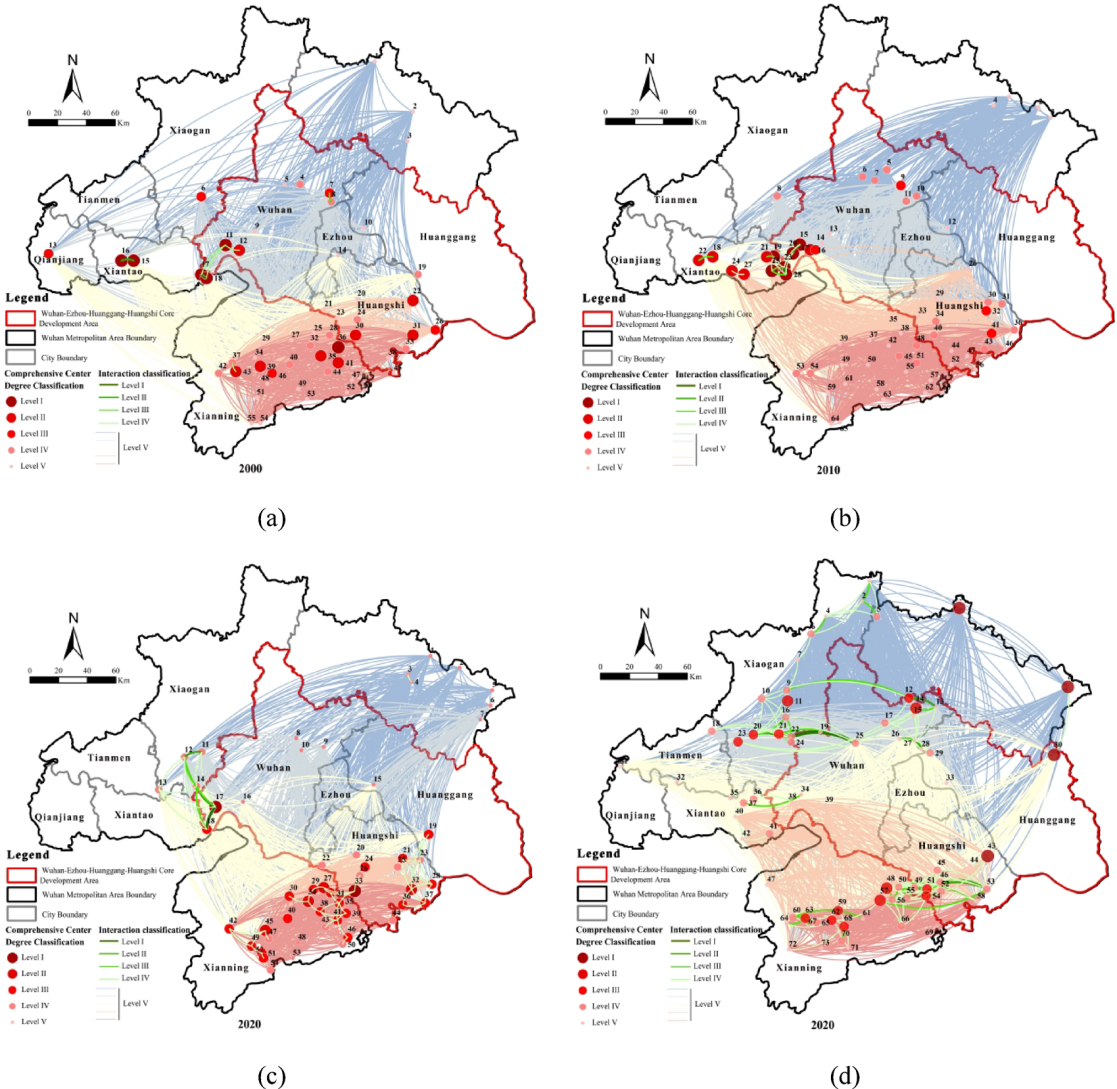
Spatial and temporal evolution of ecological networks: (**a**) ecological network (> 1000 ha), 2000; (**b**) ecological network (> 1000 ha), 2010; (**c**) ecological network (> 1000 ha), 2020; (**d**) ecological network (> 500 ha), 2020. Maps in this figure were created by the authors using ArcGIS 10.8.1 (Esri, Redlands, CA, USA; https://www.esri.com) and further refined in Adobe Photoshop 2024 (Adobe Inc., San Jose, CA, USA; https://www.adobe.com).

In 2000 (Fig. 7a), the ecological network was in its initial formation stage, featuring many nodes in the south, scattered core nodes, and weak connectivity. Only seven high-interaction corridors were sporadically distributed in western and central regions, resulting in low efficiency of material, energy, and information transfer between sources. In 2010 (Fig. 7b), node numbers increased in the western and southern regions, core nodes gradually concentrated, and network connectivity improved. Thirteen high-interaction corridors in the mid-west enhanced interactions among sources and improved transmission efficiency. In 2020 (Fig. 7c), network stability increased substantially. Nodes expanded in southern and northeastern regions, core nodes clustered in the south, connections became closer, and overall network structure became more efficient. Fifty-one high-interaction corridors concentrated in the south and mid-west, significantly enhancing source interactions and transmission efficiency. In the 2020 ecological network including sources larger than 500 ha (Fig. 7d), key core nodes were concentrated at Zhuhu Wetland, Futianhe Town, Dabie Mountain, Wanghu Wetland, Ganhe Water Reserve, and Qianshan Forest Park. These nodes formed densely interconnected hubs, constituting the structural backbone of the network. Interaction intensity among Xiaogan, central Wuhan, Xianning, and Huangshi increased significantly, emphasizing critical connectivity dynamics essential for network resilience.

In summary, the Wuhan Metropolitan Area’s ecological network evolved from initial scattered cores and weak connectivity to a stable, clustered, and efficient structure. Core nodes concentrated in the south, high-interaction corridors expanded, and global transmission efficiency remained high despite minor fluctuations. Key nodes formed structural backbones, highlighting priority areas for maintaining network resilience.

### Resilience of the ecological network in the Wuhan metropolitan area

#### Resilience changes under node disturbance scenarios

Between 2000 and 2020, ecological network resilience in the Wuhan Metropolitan Area exhibited distinct responses under probabilistic and deterministic attack scenarios. Overall, ecological strategies promoted gradual optimization, balancing efficiency and redundancy, and shifting the network’s development from incremental expansion to improved quality and efficiency.

**Fig. 8:**
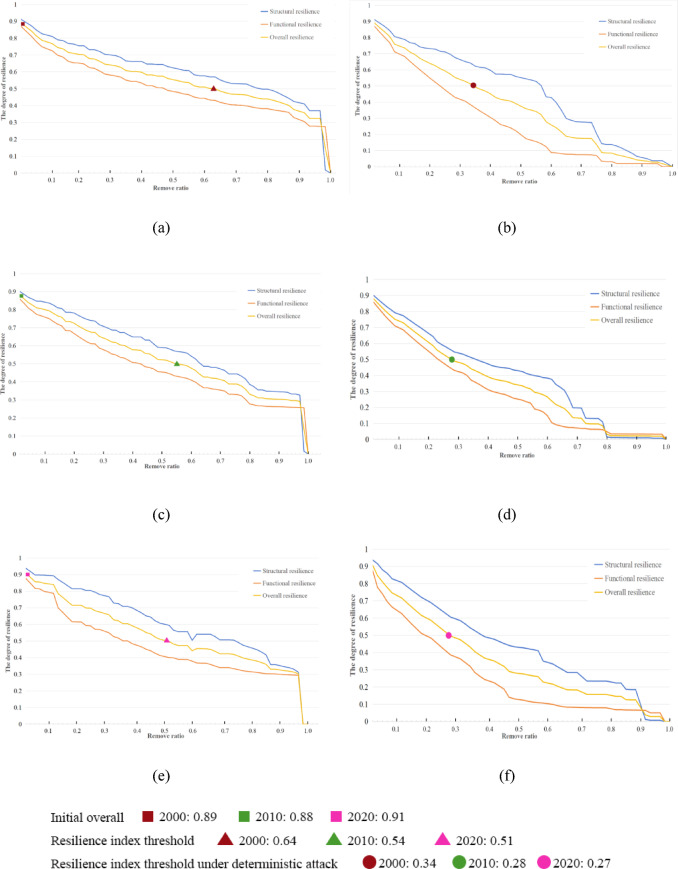
Resilience changes under node disturbance scenarios: (**a**) Under the probabilistic attack scenario in 2000; (**b**) Under the deterministic attack scenario in 2000; (**c**) Under the probabilistic attack scenario in 2010; (**d**) Under the deterministic attack scenario in 2010; (**e**) Under the probabilistic attack scenario in 2020; (**f**) Under the deterministic attack scenario in 2020. Figures were generated by the authors using Python 3.9 (Python Software Foundation, https://www.python.org), and visualized in Microsoft Excel 2019 (Microsoft Corporation, Redmond, WA, USA; https://www.microsoft.com).”

Under probabilistic attack scenarios, the resilience trend from 2000 to 2020 was generally consistent (Fig. 8). Resilience declined slowly when node removal rates ranged from 0% to 90%, decreased sharply between 90% and 98%, and approached zero above 98%, indicating near-complete network collapse. The resilience index threshold—defined as the node removal rate at which overall resilience falls to half its initial value—was 0.64 in 2000 (Fig. 8a), 0.54 in 2010 (Fig. 8c), and 0.51 in 2020 (Fig. 8e). Initial overall resilience first declined and then increased, with values of 0.89, 0.88, and 0.91. Although the threshold showed a downward trend, its decline slowed from 2010 to 2020, reflecting gradual optimization of network resilience. Despite the substantial impacts of urbanization and climate change, ecological strategies, including the “Yangtze River Protection Initiative,” contributed to measurable improvements in network resilience.

Under deterministic attacks, network resilience declined more rapidly, highlighting the critical role of core ecological nodes in maintaining network stability (Fig. 8). Between 2000 and 2020, node removal rates causing resilience to fall to half were 0.34 (2000), 0.28 (2010), and 0.27 (2020), significantly lower than thresholds under probabilistic attacks. This indicates the increasing control of core nodes over network stability. Notably, in 2010, removing more than 80% of nodes caused overall resilience to approach zero, whereas in 2020, more than 90% removal was required. This suggests that in the early stage, the network prioritized key node efficiency at the expense of resistance to deterministic attacks. During the later stage, redundancy was incorporated alongside efficiency, leading to optimized network resilience.

Structurally, as node removal rates increased, structural resilience remained relatively stable, whereas functional resilience followed a “rapid decline—stabilization—sharp decline” pattern. Under probabilistic attacks, the network reached a “stable” state when node removal rates ranged from 80% to 95%, with functional resilience values of 0.28, 0.26, and 0.30 in 2000, 2010, and 2020, respectively. Under deterministic attacks, the “stable” state occurred at node removal rates of 60%–80%, with functional resilience values of 0.08, 0.07, and 0.08 in 2000, 2010, and 2020. Functional resilience initially decreased and later increased, reflecting optimized network redundancy during the later stage.

#### Impact scope analysis of hub node disruption

We analyzed degree, closeness, and betweenness centrality of the 2020 ecological network (> 500 ha) using UCINET. Targeted attacks on high-centrality nodes revealed variations in network stability. Corridors were classified into critical (Levels 1–4) and general (Level 5) to assess connectivity resilience under centrality-based disturbances.

**Fig. 9:**
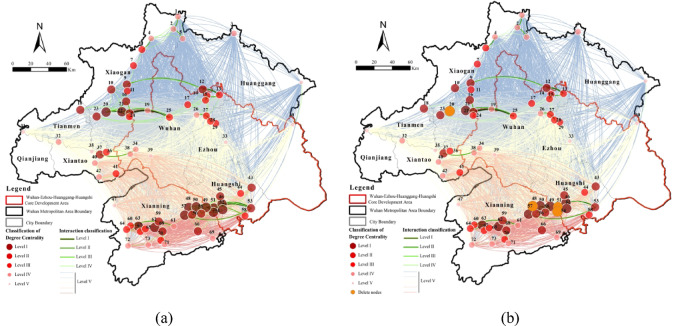
Network dynamics under targeted removal of hub nodes based on degree centrality: (**a**) Normal situation; (**b**) Remove the top 5% degree centrality nodes. Maps in this figure were created by the authors using ArcGIS 10.8.1 (Esri, Redlands, CA, USA; https://www.esri.com) and further refined in Adobe Photoshop 2024 (Adobe Inc., San Jose, CA, USA; https://www.adobe.com).

Degree centrality measures a node’s influence within the ecological network, identifying core ecological sources. High-degree nodes were concentrated in southern Xianning, northwestern Xiaogan, and the Xianning-Huangshi border (Fig. 9). Key hubs, including Laoguan Lake Wetland Park, Xiandao Lake Scenic Area, Fushui Reservoir, and Damu Mountain Forest Park, govern overall connectivity. Their targeted disruption eliminated 31 critical corridors (31.31% of high-priority links), mainly affecting northwestern Xiaogan wetlands and the Xianning-Huangshi interface. Secondary, tertiary, and quaternary corridors decreased by 4, 7, and 20, respectively, reducing ecosystem functionality and resilience, highlighting the irreplaceable role of high-degree nodes.

**Fig. 10:**
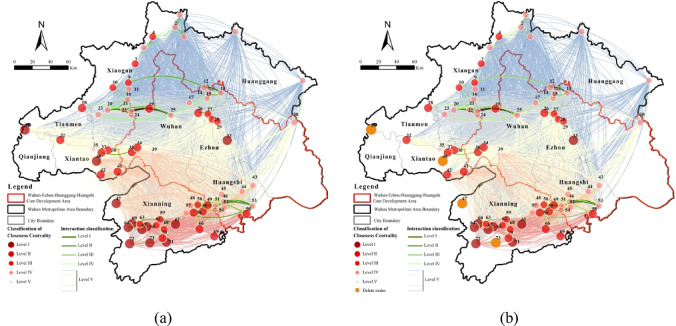
Network dynamics under targeted removal of hub nodes based on closeness centrality: (**a**) Normal situation; (**b**) Remove the top 5% closeness centrality nodes. Maps in this figure were created by the authors using ArcGIS 10.8.1 (Esri, Redlands, CA, USA; https://www.esri.com) and further refined in Adobe Photoshop 2024 (Adobe Inc., San Jose, CA, USA; https://www.adobe.com)

Closeness centrality quantifies a node’s independence and ability to connect other nodes without intermediaries. High-closeness nodes were clustered in central Xianning, with secondary concentrations in Qianjiang, Xiantao, and Huanggang (Fig. 10). Key nodes include Node 47 (Dayan Lake, Xianning), Node 31 (Gaoshibei Town, Qianjiang), Node 40 (Pengchang Town), and Node 73 (Qingshan Reservoir, Chongyang). Disruption of these nodes eliminated 1 critical and 281 general corridors, primarily impacting central-western Tianmen, central-western Xiantao, and northern Xianning.

**Fig. 11:**
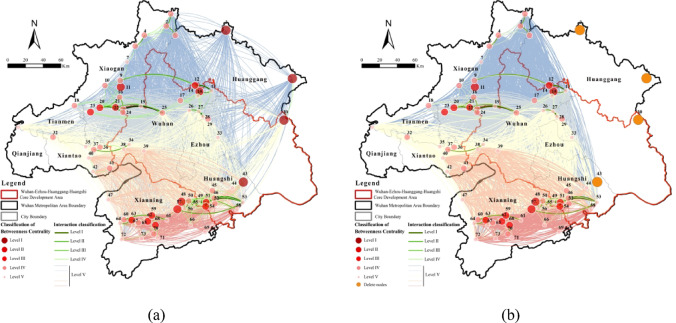
Network dynamics under targeted removal of hub nodes based on betweenness centrality: (**a**) Normal situation; (**b**) Remove the top 5% betweenness centrality nodes. Maps in this figure were created by the authors using ArcGIS 10.8.1 (Esri, Redlands, CA, USA; https://www.esri.com) and further refined in Adobe Photoshop 2024 (Adobe Inc., San Jose, CA, USA; https://www.adobe.com).

Betweenness centrality measures a node’s spatial mediation and bridging capacity within the ecological network. High-betweenness nodes were concentrated in southern Xiaogan, central Wuhan, central-eastern Xianning, and southwestern Huangshi (Fig. 11). Key mediators include Node 3 (Futianhe Town, Huanggang), Node 8 (Caopandi Town, Huanggang), Node 30 (Yingshan County, Huanggang), and Node 43 (Wanghu Wetland, Huangshi). Notably, 10 nodes had zero betweenness centrality, indicating that 13.3% of nodes lack intermediary roles. Targeted removal of these four critical nodes eliminated three key corridors in southeastern Huangshi and 279 general corridors. Although small in size, these nodes act as essential “stepping stones” for species migration and ecological flow, with disruptions affecting extensive areas in the eastern Wuhan Metropolitan Region.

In summary, high-centrality nodes are essential for maintaining regional connectivity and ecosystem stability. Degree-centrality nodes, concentrated in southern Xianning, northwestern Xiaogan, and the Xianning-Huangshi border, control primary corridors. Closeness-centrality nodes, mainly in central Xianning, Qianjiang, Xiantao, and Huanggang, influence intermediate connectivity. Betweenness-centrality nodes in southern Xiaogan, central Wuhan, central-eastern Xianning, and southwestern Huangshi serve as “stepping stones” for species migration. Damage to these hubs significantly reduces ecological network resilience, emphasizing the need for targeted conservation strategies.

## Discussion

### Spatiotemporal evolution and driving mechanisms of ecological source areas

From 2000 to 2020, the number of ecological source areas in the Wuhan Metropolitan Area first increased and then decreased, showing a clear time-series pattern. Spatially, these ecological sources evolved from scattered patches to concentrated, contiguous clusters, while core nodes increasingly exerted global control over the network. This transformation was closely driven by phased policy interventions and the gradual restoration of natural substrates. During the early stage, projects such as the “Grain-for-Green Program” and the “Yangtze River Shelter Forest Program” promoted the expansion of ecological source areas. However, most newly added sources were located in ecologically vulnerable or peripheral areas, forming a scattered pattern, as exemplified by wetland restoration in the Jianghan Plain. In the later stage, the “Ecological Red Line” policy and the “Yangtze River Great Protection” strategy facilitated the integration of ecological spaces. Peripheral barrier areas formed by the Dabie and Mufu Mountains, benefiting from superior natural substrates, developed core-node clusters through contiguous protection of ecological sources. In the central plain, expansion of construction land led to the disappearance of some small-scale ecological sources, further reinforcing the pattern of “periphery-concentrated, central-sparse” distribution. Although core-node concentration enhanced network transmission efficiency, it also reduced redundancy, highlighting potential vulnerability. This structural arrangement poses systemic risks under targeted attacks, consistent with the efficiency-robustness trade-off theory in complex networks proposed by Albert et al.^8^.

Notably, in 2000, ecological sources were sparse and scattered along the Wuhan–Xiantao border region. By 2010, ecological sources had expanded, and by 2020, they coalesced into a contiguous cluster, forming a key ecological source group in the central-western Wuhan Metropolitan Area. This evolution reflects the combined influence of cross-regional governance and the synergistic effect of natural substrates. The Wuhan–Xiantao border lies in the heart of the Jianghan Plain, characterized by a dense network of rivers and lakes and interlaced natural wetlands and farmlands, providing a high-quality natural substrate. Rapid urbanization around 2000 caused fragmentation of ecological sources. In 2012, the urban agglomeration plan for the middle Yangtze River stipulated joint construction of the “Jianghan Plain Ecological Corridor” by Wuhan and Xiantao. In 2016, under the “Yangtze River Great Protection” strategy, ecological red lines in Hannan District (Wuhan) and Pengchang Town (Xiantao) were integrated contiguously. Scattered wetlands and farmland shelter forests were unified under coordinated management, leading to the establishment of the “Wuhan–Xiantao Dongjing River Ecological Corridor”. Illegal docks were removed, ecological bridges constructed, and barriers between sources eliminated. Simultaneously, Pengchang Town’s non-woven fabric industry transformed into “green intelligent manufacturing,” and vacated industrial land was converted into ecological nodes, forming a concentrated, contiguous ecological group connected with the Hannan smart agriculture demonstration area in Wuhan.

### Spatial differentiation and efficiency enhancement of ecological corridors

Ecological corridors exhibited a spatial pattern characterized by high density in the south and northeast and low density in the north and central regions. Although the total number of corridors declined, the intensity of interactions among remaining corridors increased markedly. In the south and northeast, superior natural substrates combined with policy protection fostered the formation of a dense primary corridor network. In contrast, the central-western plain experienced pronounced corridor fragmentation due to road network expansion and encroachment by cultivated land. Notably, while the total number of corridors decreased, the proportion of primary corridors increased substantially. This trend suggests that optimizing corridor quality substantially enhanced species movement efficiency. This pattern aligns with the “hierarchical optimization” theory proposed by De Montis et al.^67^. Specifically, ecological restoration projects can achieve higher ecological flow efficiency using fewer pathways by enhancing connectivity of key corridors.

Notably, the interaction strength between the Ganhe Water Source (Xianning City) and Wanghu Wetland (Huangshi City) increased markedly, establishing this corridor as the core passage of the southern ecological network in the Wuhan Metropolitan Area. The corridor connecting Xianning and Huangshi traverses the industrial concentration zone in Xianning. Generally, anthropogenic activities, particularly industrial production, can disrupt ecological corridors. However, in this corridor, the “ecological isolation zone” policy—including ecological ditch construction and restriction of industrial land expansion—successfully maintained connectivity. This case demonstrates the feasibility of the “economic-ecological synergy path” proposed by Zeng et al.^49^. In other words, refined spatial management and control can balance ecological efficiency and economic development in industrial-intensive regions.

### Trade-off between ecological network efficiency and resilience

The ecological network of the Wuhan Metropolitan Area exhibits both synergy and inherent tension between scale-free and small-world characteristics, indicating a pronounced trade-off between efficiency and resilience. This tension not only drives structural optimization and network upgrading but also highlights the fundamental challenge of balancing “efficient transmission” with “disturbance-resistant stability” in ecological governance.

From the perspective of network evolution, both network density and clustering coefficient increased substantially, with core nodes concentrated in the southern Xianning-Huangshi region. This pattern exhibits typical scale-free network characteristics^34^, where a few core nodes dominate ecological flow through dense connections, significantly enhancing material and energy exchange efficiency. However, this efficiency-focused structure introduces vulnerability: over-concentration of core nodes (e.g., the Xianning-Huangshi border) reduces network redundancy, causing resilience to decline sharply under targeted attacks, confirming the high sensitivity of scale-free networks to core node disruption. Moreover, anthropogenic centralization of core nodes further amplifies this vulnerability. Conversely, general nodes—including small wetlands and farmland shelterbelts in the Jianghan Plain—are widely distributed in suburban fringes. They form locally dense connections and maintain short-path links with core nodes, thereby exhibiting small-world network characteristics^33^. This configuration facilitates efficient local ecological flows and exhibits higher resilience under random attacks, highlighting the role of general nodes as the redundancy base of the network.

In summary, the trade-off between efficiency and resilience in the Wuhan Metropolitan Area’s ecological network reflects a dynamic balance between the “efficient-vulnerable” properties of scale-free nodes and the “redundant-robust” properties of small-world nodes. In the future, enhancing the disturbance resistance of core nodes should be coupled with strengthening redundancy by densifying secondary corridors among general nodes. This approach achieves synergy between scale-free and small-world characteristics, thereby establishing an ecological network that combines efficient transmission with robust resilience.

### Implications for regional ecological governance

This study systematically analyzed the spatial evolution and structural characteristics of ecological security patterns in the Wuhan Metropolitan Area, integrating data on ecological sources, corridors, and network resilience. At the node level, high-value ecological patches in the Dabie Mountains, Mufu Mountains, and major wetlands function as key hubs for maintaining ecological integrity and biodiversity. Effective protection of these strategic nodes is essential to sustain overall network stability. At the corridor level, spatial modeling shows that ecological flows are concentrated along the Yangtze River and wetland systems. However, urban expansion and infrastructure development have fragmented several critical corridors, indicating the necessity of restoration and artificial linkages. At the network level, temporal increases in connectivity, clustering, and resilience reflect gradual optimization. Nonetheless, peripheral regions remain vulnerable due to weak node interactions and limited redundancy.

Overall, these results underscore the importance of a multi-level governance framework that reinforces source protection, enhances corridor connectivity, and fosters adaptive network resilience. The subsequent section outlines targeted optimization strategies to address these challenges and promote ecological sustainability in rapidly urbanizing metropolitan areas.

### Optimization strategies for the ecological security pattern

**Fig. 14:**
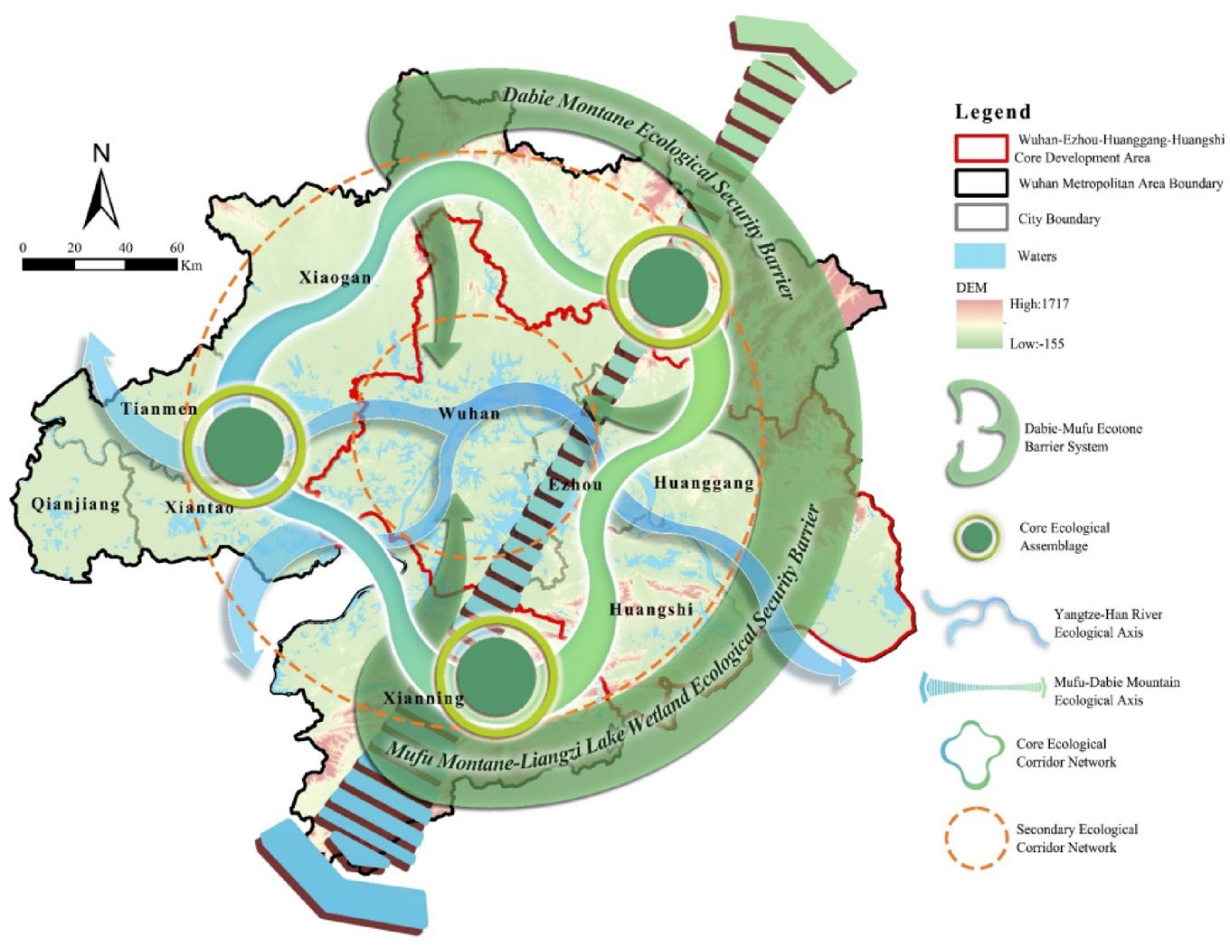
Ecological protection framework. Maps in this figure were created by the authors using ArcGIS 10.8.1 (Esri, Redlands, CA, USA; https://www.esri.com) and further refined in Adobe Photoshop 2024 (Adobe Inc., San Jose, CA, USA; https://www.adobe.com ).

1. Nodes are fundamental for maintaining ecological network stability and connectivity. Their number, contribution, and interaction strength critically influence network complexity. Based on comprehensive importance evaluation, ecological nodes are classified into core nodes, secondary nodes, and general nodes (Fig. 12).
  Eight core ecological nodes act as “super nodes,” responsible for global connectivity and material-energy transmission. They are concentrated in southern Xianning, Huangshi, and the Dabie Mountains–Mufu Mountains ecological barrier. Protection strategies combine rigid management, intelligent monitoring, and functional restoration, including: strict ecological red line zones to prohibit development; satellite and drone-based monitoring of vegetation; dredging degraded wetlands to restore hydrology; and planting diverse aquatic species to reconstruct habitats.
  Thirty-six secondary nodes function as key connectors between core and general nodes, widely distributed across the metropolitan area. While concentrated in ecologically sound southwestern and southern regions, they also occupy vulnerable areas such as the Jianghan Plain. Protecting these nodes mitigates the “dense south–sparse north” imbalance. Strategies focus on enhancing connectivity: establishing ecological buffer zones, removing illegal constructions, and, for urban fringe nodes (e.g., Wuhan-Xiantao wetlands), creating ecological islands with soil mounding, slopes, and shallow water zones to support bird roosting and fish breeding, reducing urbanization pressures.
  Twenty-nine general nodes, comprising 40% of all nodes, serve as stepping stones for biological migration. They are widely scattered across central-western plains and urban fringes, providing a redundant base that stabilizes network functions under external disturbances. Protection emphasizes natural restoration and community participation, including near-natural vegetation replanting and resident engagement via an ecological credit system to reduce anthropogenic impacts.
  **Fig. 12:**
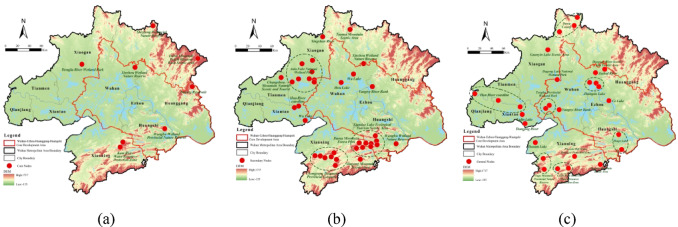
Node protection strategies: (**a**) core nodes distribution; (**b**) secondary nodes distribution; (**c**) general nodes distribution. Maps in this figure were created by the authors using ArcGIS 10.8.1 (Esri, Redlands, CA, USA; https://www.esri.com) and further refined in Adobe Photoshop 2024 (Adobe Inc., San Jose, CA, USA; https://www.adobe.com).
2. Ecological corridors act as key channels for ecological flows between source areas. Their function and network role vary depending on node interaction intensity. Based on these interactions, corridors are classified as important or general (Fig. 13).
  Eighty-six important corridors are primarily located in the southern, western, and northern metropolitan areas. In the southwest, connected corridors form efficient migration paths, whereas the northeast shows sparse and fragmented corridors, weakening ecological flows. As the network backbone, they support cross-regional material-energy transmission, biological migration, and water conservation. Damage or disruption could sever material flows between core nodes, threatening network stability. Restoration strategies involve barrier removal and function enhancement, including ecological overpasses/tunnels in industrial zones (Xianning-Huangshi), revetment transformation along the Yangtze and Han Rivers, removal of hard embankments, vegetation buffer reconstruction, and continuous subwater-riparian vegetation belts.
  General corridors, comprising over 90% of all corridors, cover most metropolitan areas and connect small, scattered ecological sources. They provide local ecological services and network redundancy, enhancing resistance to disturbances and serving as reserve channels for future expansion. Restoration focuses on redundancy assurance and monitoring, including ecological ditches in farmland-urban transition zones to link scattered wetlands and water bodies, and integrating high-standard farmland with shrub belts to form farmland-forest-water composite corridors, improving local ecological flow efficiency.
  **Fig. 13:**
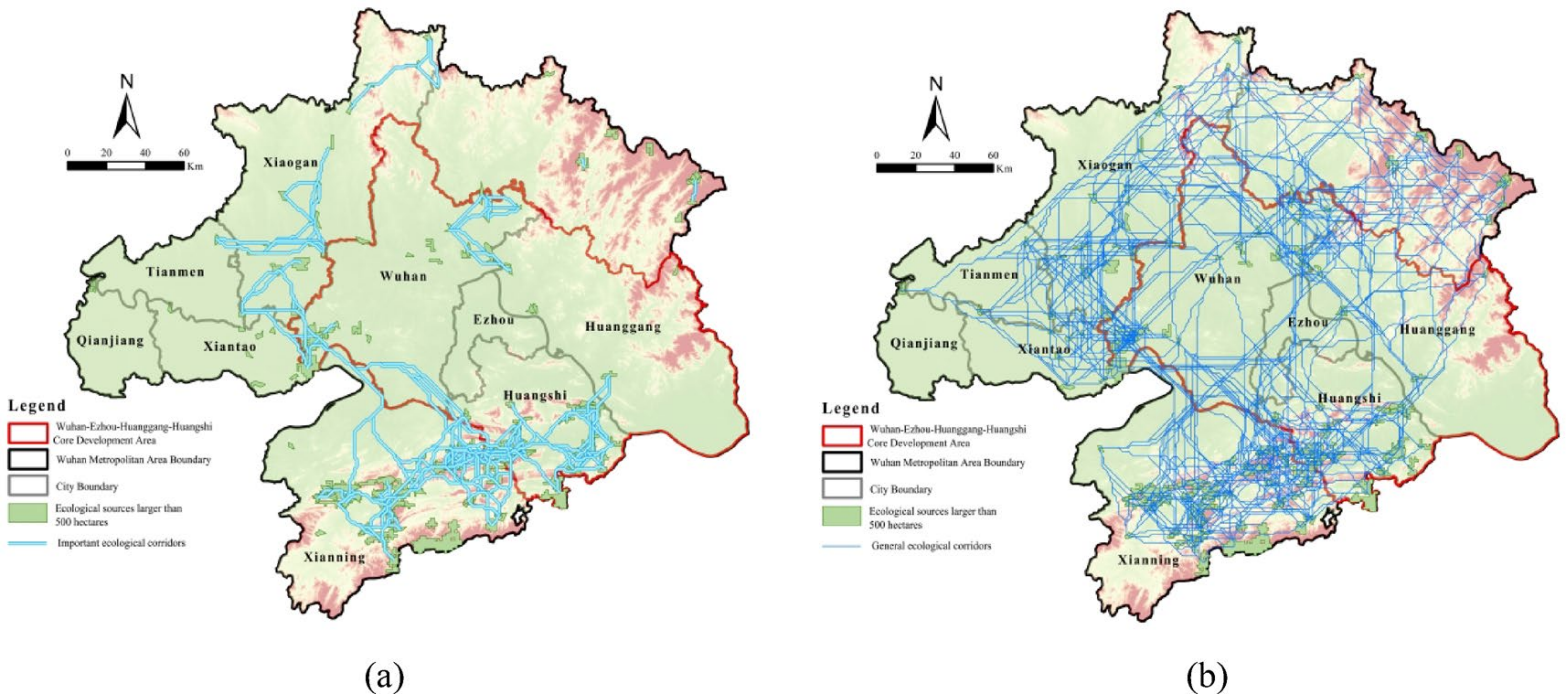
Node protection strategies: (**a**) key corridors distribution; (**b**) general corridors distribution.
3. The ecological framework of the Wuhan Metropolitan Area comprises “one screen, three cores, three axes, and multiple networks” (Fig. 14). It is developed based on a systematic analysis of the spatial distribution of the natural ecological base, ecological nodes, and corridors. Maps in this figure were created by the authors using ArcGIS 10.8.1 (Esri, Redlands, CA, USA; https://www.esri.com) and further refined in Adobe Photoshop 2024 (Adobe Inc., San Jose, CA, USA; https://www.adobe.com).

“One screen” denotes the ecological barrier formed by the Dabie Mountains–Mufu Mountains–Liangzi Lake wetland complex. This area underpins the ecological security of the metropolitan region, concentrating numerous core nodes and important corridors. It plays a central role in water source conservation, biodiversity protection, and soil and water preservation. “Three cores” include: (1) the southern core, based on the Mufu Mountain water source and Liangzi Lake wetlands; (2) the northern core, formed by the Dabie Mountain forest belt and Han River farmland shelter forests; and (3) the western core, comprising the Hanbei and Han river networks and scattered wetlands such as Chenhu and Xiaoyanhu Lakes. As the ecological skeleton of the metropolitan area, “one screen and three cores” critically influences network stability. Strict spatial management is required, with designation as core ecological protection red lines. Development and construction within these zones should be prohibited to prevent degradation of ecological functions. Core nodes are decisive for network connectivity. To mitigate vulnerability from node over-concentration, redundant corridors such as shelter forests should be arranged around core nodes to provide alternative pathways and enhance network robustness.

“Three axes” include the Yangtze River main axis, the Han River secondary axis, and the Mufu–Dabie Mountains longitudinal axis. “Multiple networks” comprise a multi-level redundant network: a main network of important corridors connecting the three cores to maintain efficiency, and a secondary network of general corridors to ensure redundancy. The Yangtze and Han River axes traverse densely populated areas. Protection strategies include restricting development intensity, establishing ecological buffer zones, and reconnecting fragmented corridors via ecological ditches to achieve economic-ecological synergy. Ecological flow between the Mufu and Dabie Mountains is currently weak. Densification of northeastern mountain forest corridors and removal of barriers between the Dabie and Mufu Mountains will gradually form the Mufu–Dabie longitudinal axis, enhance important corridor distribution, create a metropolitan ring of key corridors, and improve ecological flow efficiency. Although ecological flows in general corridors are weaker, they hold strategic value as flexible spaces for climate adaptation and urban expansion. Policy guidance should enhance ecological context, reserve corridor spaces, and strengthen the secondary network to maintain redundancy.

Strengthening the ecological base through “one screen and three cores” and balancing network efficiency and resilience via “three axes and multiple networks” can enhance ecological security and network resilience in the Wuhan Metropolitan Area. This framework offers a reference for coordinated ecological protection and sustainable development within the Yangtze River Economic Belt.

### Limitations and future research directions

Although this study provides an in-depth analysis of the ecological network resilience in the Wuhan Metropolitan Area, several limitations remain that warrant further investigation. First, the simulation of sudden events is limited to two categories: “random failures” and “deliberate attacks.” It lacks targeted analysis of specific risks, including extreme climatic events and geological disasters. Future research should develop a multi-scenario coupling model to simulate the impacts of compound risks on the ecological network and to design differentiated resilience-enhancing strategies. Second, various spatial quantification indicators exist for identifying ecological source areas and extracting corridors, such as the landscape vulnerability index and the landscape safety adjacency index. These indicators could be applied in future studies to obtain more practical and actionable insights. Additionally, the construction of the ecological security pattern in this study relied on some relatively static indicators due to data limitations. Future research should incorporate dynamic indicators to enhance the scientific rigor of the study. Finally, this study focuses on the Wuhan Metropolitan Area and does not examine its linkages with the broader urban agglomeration in the middle reaches of the Yangtze River or the national ecological security pattern. This overlooks the significance of cross-regional ecological flows and coordinated governance. Future studies should address cross-regional coordination to improve ecological network integration at broader spatial scales.

## Conclusion

This study evaluates the ecological network resilience of the Wuhan Metropolitan Area from 2000 to 2020 from the perspective of ecological security patterns. The results indicate that: (1) the number of ecological source areas exhibits a rise-then-decline trend, with spatial distribution shifting from fragmentation in the early stage to agglomeration in the later stage, accompanied by gradual concentration of core nodes enhancing overall network control; (2) ecological corridors show a spatial pattern of higher density in the south and periphery and lower density in the north and central areas; although the total number of corridors decreases, interaction intensity and species migration efficiency increase; (3) the ecological network evolves from incremental expansion toward improved quality and efficiency, gradually forming a more integrated and stable structure.

Compared with previous studies, this research offers three main contributions. First, it transcends traditional administrative boundaries by selecting the Wuhan Metropolitan Area as the research unit, encompassing diverse geographic environments including the Jianghan Plain, the Dabie Mountain region, and the Yangtze River mainstream. Second, it integrates multi-dimensional data from natural, social, and economic domains, incorporating novel evaluation indicators such as the nighttime light index and PM2.5 concentrations. Finally, using multi-temporal data from 2000, 2010, and 2020, it applies disturbance scenario simulations to evaluate network resilience, enabling dynamic tracking of ecological network evolution and providing insights into the impacts of human activities and environmental changes.

Theoretically, the study advances understanding of the dynamic evolution of ecological network resilience under urbanization and climate change. It clarifies the trade-off between efficiency and robustness in complex ecological networks and offers a theoretical reference for investigating cross-regional dynamic response mechanisms. Practically, hierarchical node protection and corridor quality improvement provide relevant strategies to mitigate ecological fragmentation associated with administrative boundaries. Additionally, the approach combining multi-source data integration with long-term dynamic simulations offers methodological support for refined ecological assessments and the development of adaptive governance policies. In summary, by integrating theoretical frameworks, practical strategies, and methodological approaches, this study provides reference frameworks for promoting sustainable development and ecological security pattern planning in ecologically sensitive and rapidly urbanizing regions.

## Data Availability

Data will be made available and if you wish to access it, please contact the corresponding author.
